# Diel Vertical Migration in Deep Sea Plankton Is Finely Tuned to Latitudinal and Seasonal Day Length

**DOI:** 10.1371/journal.pone.0064435

**Published:** 2013-05-22

**Authors:** Hans van Haren, Tanya J. Compton

**Affiliations:** Royal Netherlands Institute for Sea Research (NIOZ), Den Burg, The Netherlands; Bangor University, United Kingdom

## Abstract

Diel vertical migration (DVM) is a ubiquitous phenomenon in marine and freshwater plankton communities. Most commonly, plankton migrate to surface waters at dusk and return to deeper waters at dawn. Up until recently, it was thought that DVM was triggered by a relative change in visible light intensity. However, evidence has shown that DVM also occurs in the deep sea where no direct and background sunlight penetrates. To identify whether such DVM is associated with latitudinal and seasonal day light variation, one and a half years of recorded acoustic data, a measure of zooplankton abundance and movement, were examined. Acoustic Doppler current profilers, moored at eight different sub-tropical latitudes in the North-Atlantic Ocean, measured in the vertical range of 500–1600 m. DVM was observed to follow day length variation with a change in season and latitude at all depths. DVM followed the rhythm of local sunrise and sunset precisely between 500 and 650 m. It continued below 650 m, where the deepest penetrable irradiance level are <10^−7^ times their near-surface values, but plankton shortened their time at depth by up to about 63% at 1600 m. This suggests light was no longer a cue for DVM. This trend stayed consistent both across latitudes and between the different seasons. It is hypothesized that another mechanism, rather than light, viz. a precise biochemical clock could maintain the solar diurnal and seasonal rhythms in deep sea plankton motions. In accordance with this hypothesis, the deepest plankton were consistently the first to migrate upwards.

## Introduction

Diel vertical migration (DVM) in ocean zooplankton is likely to represent the largest daily migration of animals on earth, in terms of biomass [1]. Most commonly, plankton migrate to surface waters at dusk and return to deeper waters at dawn. This pattern of migration is considered to reflect a trade-off between to need to feed versus predator avoidance, especially in the sunlit photic zone [2], [3].

Knowledge of DVM in plankton goes back centuries (e.g., [4], [5]). But since the advance of acoustic methods since the early 1980s, considerable details about DVM have been revealed; and have also been verified with plankton net-tows (e.g., [6], [7]). Acoustics provide numerous advantages over plankton net-tows. They are non-invasive, they can detect the larger zooplankton capable of swimming out of the way of nets, they can be used for continuous monitoring [8] and they can directly measure the vertical speed of zooplankton [9]. Consequently, acoustic backscattering is now a common method used to monitor relative zooplankton abundance and their vertical migrations (e.g., [6], [10]–[11]). But acoustics are limited in that they cannot provide taxonomic resolution and they have difficulty in quantifying plankton biomass with a single frequency instrument [12].

In the photic zone, down to a few 100 m from the surface, temporal high-resolution acoustic data, representing plankton migration, identified different stages of dusk ascent and dawn ascent [13]. Ascent started slowly well before sunset but the fast portion ended before twilight. The first slow descent started hours before twilight, while the fast descent ended just before sunrise. This confirmed earlier conclusions that near-surface DVM was triggered by local solar variations. Furthermore, near-surface evidence using annual time series from acoustics in high latitude sites proved seasonal variations in day length estimated from DVM [11], [14].

Initially, DVM seemed limited to the photic zone. But in the arctic, synchronized DVM behaviour of zooplankton was found in both open and ice-covered waters of 30–60 m, where light was below the detection level of many standard irradiance meters [15]. Interestingly, this migration was linked with background light intensity levels at less than half the minimum detection level for the human eye. Deep acoustic observations down to 1200 m [9] and down to 1400 m [16] have also demonstrated DVM. But by contrast with the Arctic, deep sea migration is below both visible and background light intensities. At these depths, in the subtropics, a 1% light level reaches ∼100 m deep, whereas the deepest penetrable 480 nm irradiance level is <10^−7^ times the near-surface value at 600 m [17]. A 1.5 years long time series from the Canary Basin [16] even demonstrated a lunar monthly modulation of DVM, exactly in phase with the moon. Moonlight reaches its <1‰ light level at about 125 m in that area [17]. Thus a gap in our knowledge remains on whether DVM in the deep sea is pervasive across latitudes and whether the timing of migration is sensitive to changes in seasonal day length.

To identify whether DVM from the deep-sea is associated with seasonal day light variation across latitudes, records of one and a half years of acoustic data, a measure of zooplankton migration, were examined. The acoustic data was derived from Acoustic Doppler current profilers (ADCP) at eight different sub-tropical latitudes ranged between 500 and 1650 m vertically. More than half of this range is not reached by any detectable daylight [17].

## Materials and Methods

### ADCP deployment

Over a period of three years, up- and downward looking 75 kHz Teledyne-RDI ADCPs have been moored at eight sites between the equator and 33°N in the North-Atlantic Ocean (Table 1 for details). Record lengths varied between 17.5 and 19.5 months. The ADCPs were mounted around 1000–1600 m in the top-buoy of 3000–3700 m long moorings located in 4500–5200 m water depth in the Canary, Cape Verde and Ceará (equatorial Brazil) Basins. No specific permissions were required for these moorings. The field studies did not involve endangered or protected species, as no samples were collected.

**Table 1:** Details moored Teledyne RDI 75-kHz, 20°-beam angle Longranger ADCPs.

Name	LOC113	LOC114	LOC143	LOC163	LOC183	LOC184	LOC164	LOC144
Latitude	33°00.0′N	30°00.0′N	28°48.0′N	22°29.6′N	14°59.9′N	02°30.2′N	00°57.1′N	00°00.0′N
Longitude	022°24.4′W	023°00.0′W	023°59.6′W	027°19.0′W	030°00.3′W	038°01.6′W	037°54.3′W	036°59.5′W
Waterdepth	5274 m	5115 m	5110 m	5396 m	5390 m	4474 m	4475 m	4492 m
Deployment	10/06/2006	23/11/2007	25/05/2006	30/05/2006	01/06/2006	11/12/2007	15/12/2007	12/12/2007
Recovery	22/11/2007	11/07/2009	24/11/2007	27/11/2007	30/11/2007	29/06/2009	23/06/2009	24/06/2009
Up/down look.	Down	Up	Up	Up	Up	Up	Down	Up
Transmit length	10 m	11 m	11 m	11 m	11 m	11 m	11 m	11 m
Instrument dep.	1350 m	1350 m	1650 m	1500 m	1400 m	1003 m	1053 m	996 m
First bin	1374 m	1327 m	1627 m	1476 m	1377 m	978 m	1075 m	973 m
# bins×bin size	60×10 m	50×10 m	60×10 m	60×10 m	60×10 m	50×10 m	50×10 m	50×10 m
Ensemble per.	1800 s	900 s	1800 s	1800 s	1800 s	900 s	900 s	900 s
Mean dU×1.5 yr	24 km	121 km	134 km	−32 km	194 km	4167 km	−1193 km	−4586 km
Mean dV×1.5 yr	−1 km	3 km	172 km	−27 km	42 km	−2200 km	830 km	1572 km

U-direction is zonal (positive East), V-direction is meridional (positive North).

The ADCPs have four beams that are slanted at an angle of θ = 20° to the vertical. They were set to range 500–600 m from the head, in 10 m vertical intervals. ADCPs' pressure and tilt sensor information showed that the moorings did not move much in the Canary and Cape Verde Basins, <1.5° tilt angle implying mooring motions across <1.2 m in the vertical z and <100 m in horizontal x, y directions. In the equatorial region, a single strong current event caused deflection of about 5°. These small mooring motions are due to efforts to minimize drag using two large, elliptically shaped, 300–500 kg net buoyancy elements and a thin (0.007 m diameter) nylon-coated steel cable.

### Determination of zooplankton migration from ADCP-data

The current components (u, v, w) in the associated Cartesian coordinates (x-East, y-North, z-up) are measured at different depth levels z (usually 10 m apart) per time step t ( = 900 or 1800 s) as averages over the horizontal acoustic beam spread. This is due the θ = 20° vertical slant angle. The spread measures 20–440 m, depending on the range from the ADCP. In contrast, the acoustic reflections ‘echo intensities’ (I) are obtained per beam. They are thus averaged over much smaller horizontal scales of typically 10 m. As the I-data are dominated by the attenuation of sound through the water column, the suspended particle signal dI is obtained by subtracting the time mean, denoted by <…>, from the raw data for each z, dI(z, t) = I(z, t)−<I>(z) [dB]. Far from bottom boundaries, as found in the open North-Atlantic, the dominant source for 75 kHz ADCP dI are macroplankton, including zooplankton and ichtyoplankton, and fish species that have sizes larger than about 0.01 m [6], [9], [18]. In fact, without ocean life of this size, (75 kHz) ADCPs simply do not function in clear ocean waters.

Variations in dI and w will represent variations in the DVM of plankton. These variations consist of changes in plankton abundance, size and form (dI, [12]), and in plankton motions (w). The existence of macroplankton at 500–2000 m in the NE Atlantic Ocean have been confirmed between 15 and 53°N using net-tows and manned submersible [19], [20].

For monitoring daily variations in the relative amount of plankton (dI), composites are computed per selected monthly period (N = 30 days),
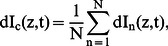
(1a)where dI_n_ represents dI at a given day n [9]. Similarly, for monitoring DVM-movements the composite w_c_ is computed from w,
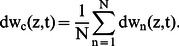
(1b)These vertical currents are dominated by internal (tidal) wave motions and by plankton migrations, although at clearly distinguishable different frequencies [16].

We thus have two independent and different measures from a single acoustic instrument for remotely studying vertical plankton migration. The daily migrations, in raw dI- and w-data or in averages over a month, as in equation (1), are used for monitoring day length variations in DVM. This is done by searching for the times of daily minima and maxima in w and in the derivative of dI at given depths. The time series of such minima and maxima are then harmonic analyzed [21] with a period of exactly one year, to determine amplitude, phase and mean. As a measure for “error”, the normalized residual variance is used. It is noted that this “error” is not a random error, as it contains all other possible harmonic motions including tides. Especially for the equatorial region the “error” is expected to be large as seasonal variations will be negligible.

### Estimation of day length

The DVM-variations in day length will be compared below with exact sunrise/sunset data for the given sites, as computed using http://aa.usno.navy.mil/data/docs/RS_OneYear.php. Latitudinal variations were computed using the model [22],
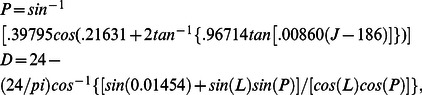
(2)where D is day length (in hours), J is day of the year and L is the latitude (in radians). This model included the effects of declination of the earth (23.5°) and the atmospheric refraction. It deviated from true solar day length by less than 1 minute for |L|<40°.

## Results

**Figure 1:**
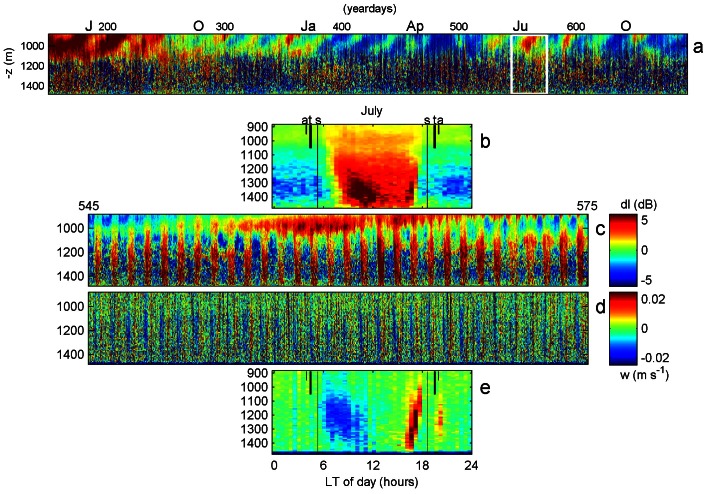
Overview of relative echo intensity data (dI), amount of plankton, and vertical current data (w), movement of plankton, from an upward looking ADCP in the sub-tropical Canary Basin (at 22.5°N, 27°W, mooring LOC163 (Table 1), ranging vertically between z = −1476 and −886 m. Below −1150 m noise is increased, due to lower amounts of plankton once they have migrated upwards after dusk. Time in 2007 is yearday +365. (a) Entire 18 months of depth versus local solar time series of plankton, or echo intensity, relative to its time mean. (b) Monthly averaged summer time data from white rectangle in a. Symbols ‘s’ indicate local sunrise and sunset, ‘t’ nautical twilight and ‘a’ astronomical twilight. (c) Month of July raw data (rectangle in a.) of which b. is the average. (d) as c., but for w. (e) as b., but for w.

All acoustic observations investigated here showed a seasonal variation, see the example from 22.5°N in Figure 1a. Superimposed on this seasonal cycle were monthly and shorter period variations. Typical variations in dI were 10 dB (Fig. 1b,c), or one order of magnitude in back-scattered sound energy, and in w 0.03 m s^−1^ (Fig. 1d,e). These speeds were maintained for about 3 hours, resulting in a vertical range of DVM of typically 300 m. The zooplankton studied here never reached the surface and most, those deeper than about 1000 m, never reached depths to where sunlight variations could be sensed.

Zooplankton at subtropical latitudes were observed over nearly the entire acoustic range, even though noise levels increased, and thus amounts of acoustic scatterers decreased, below z = −1150 m. The time series of dI did not exhibit a daily sinusoidal signal, but rather a rectangular shaped periodicity (Fig. 1b,c). In July, the range of maximum daily dI-values (dI>5 dB) had a duration of 9–11 hours within the ADCP-range (Fig. 1b). This was about 25% shorter than the sun's cycle at 22.5°N. The same was found for the daily variation in down- and upward plankton motions, when they exceeded noise levels (Fig. 1e, |w|>0.005 m s^−1^). The period of ascent (near dusk) was shorter than descent (near dawn), but associated peak values in w-amplitude, or the vertical movement of plankton, were larger for up- than for downward motion. Consequently, the vertical displacement of plankton was the same in both directions; indicating DVM.

**Figure 2:**
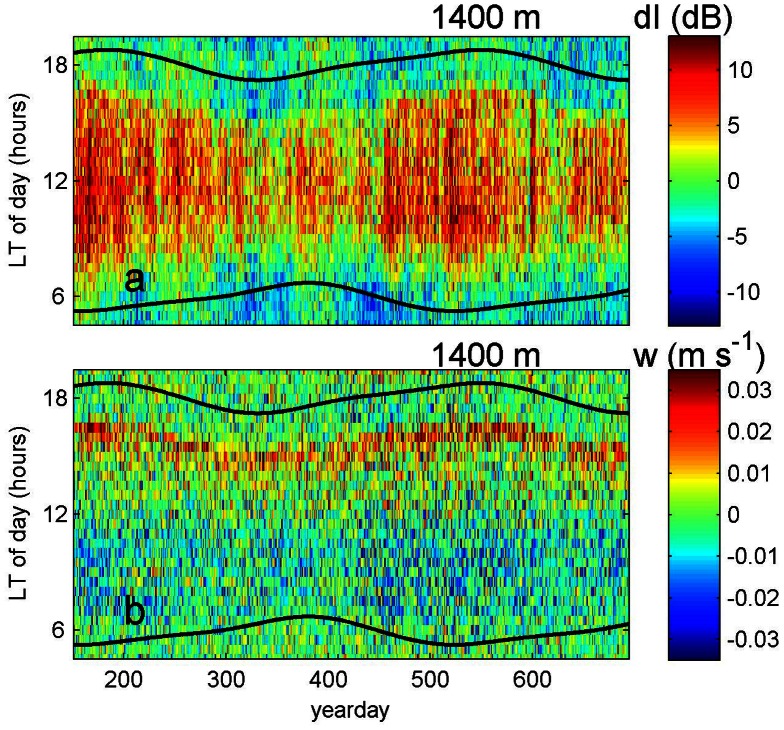
DVM-variations in day length inferred from raw data of Figure 1 at z = −1400 m, grouped in 24 hour (daily) periods. (a) Relative echo intensity. (b) Vertical current; note the much clearer upward migration of the plankton, confined in a shorter period, compared with the less clear downward migrations. In both panels, the black lines denote times of local sunrise and sunset.

**Figure 3:**
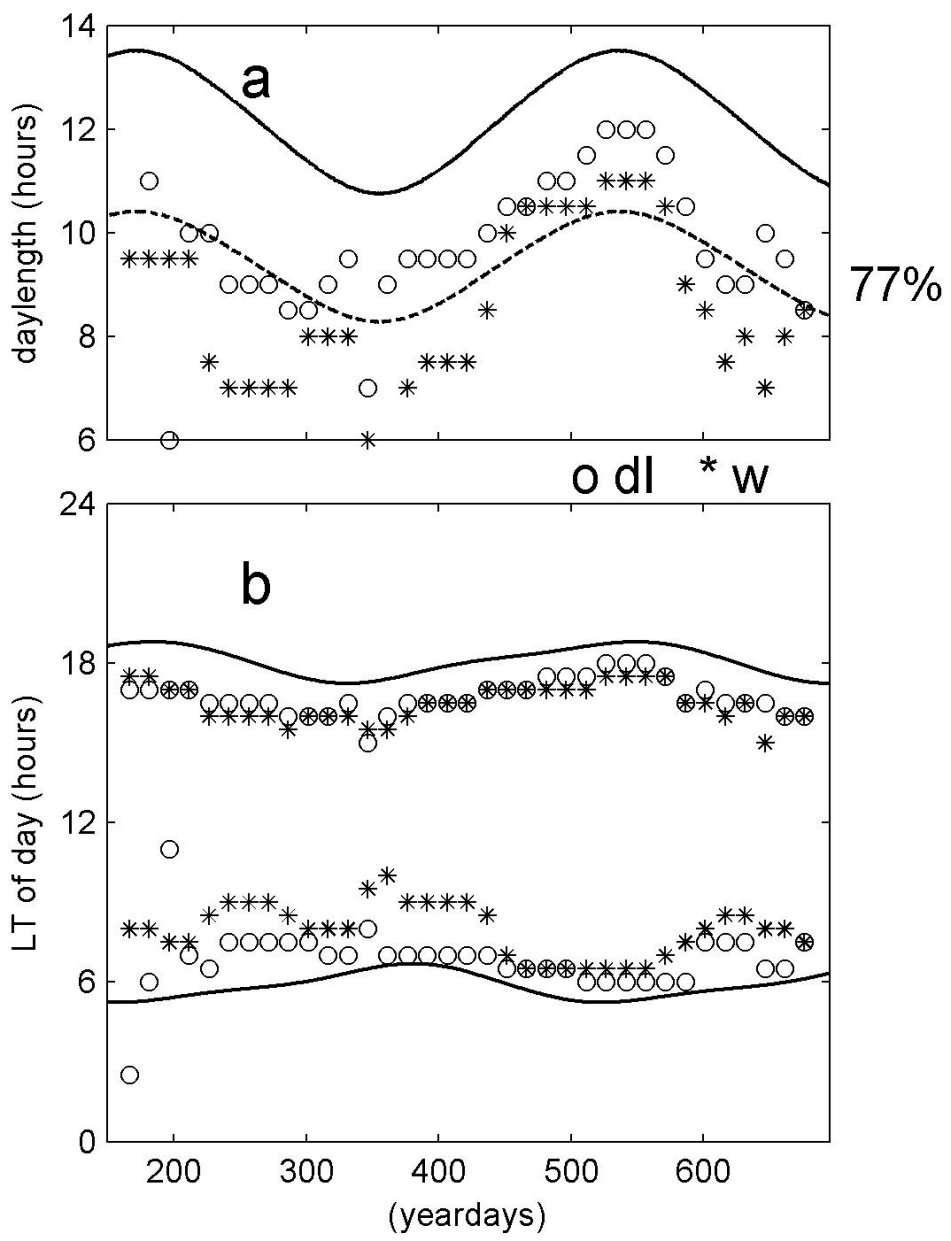
Seasonal DVM-variations in day length experienced by the plankton, as calculated in half-overlapping steps from monthly data as in Figure 1b (o) and Figure 1e (*) for z = −1021 m. Thick solid lines indicate the times of true local day length determined from the sun. (a) Total day length; dashed line is the solid line multiplied by a factor of 77%. This factor is the average day length determined from plankton DVM, being slightly larger for dI-determined day length (o) and slightly smaller for w-determined day length (*). (b) Near sunrise and sunset.

The sharply defined peak-w near sunset, as determined from daily (Fig. 2b) or monthly (Fig. 3b) averaged periods, was used to construct a time series of plankton DVM that followed seasonal sunset variation. The time series describing plankton DVM even followed the difference between spring (slow change) and autumn (rapid change), thereby explaining 75% of the variance in sunset variation at sub-tropical latitudes like 22.5°N in Figure 3. Similar observations of seasonal variation in DVM were independently made using the first derivative of dI (Fig. 3, Fig. 2a). The opposite slow/fast changes during sunrise were less well mimicked, apparently because the plankton moved in a more diffusive manner during descent. The net result of seasonal DVM-variations in day length (Fig. 3a) showed slightly larger amplitudes of plankton migration, as compared with the amplitudes of the sun's variations. However, at 1000 m the plankton seasonal day length amplitudes reflected by plankton DVM were around a mean that equaled 9.3 hours, or about 77% of the mean solar daylight period of 12.1 hours (Fig. 3a).

**Figure 4:**
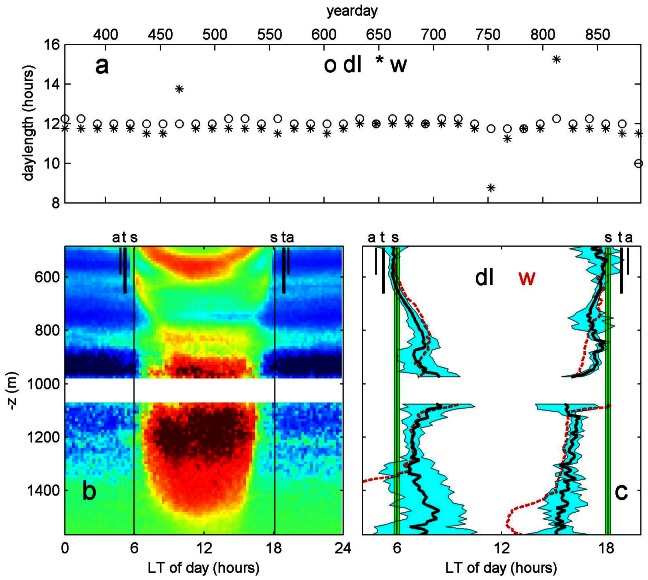
Near-equatorial observations. (a) As Figure 3a, but for z = −587 m at the equator (0°N, 37°W, mooring LOC144 (Table 1). (b) Composite mean relative echo intensity from upward looking ADCP at (0°N, 37°W) between −973 and −483 m and downward looking ADCP at (1°N, 38°W, mooring LOC164 (Table 1) between −1075 and −1565 m. (c) The associated sunrise and sunset profiles with depth with respect to the minimum morning and maximum afternoon values in dI, or plankton, variation (first derivative of, black lines). The filled blue fields indicate errors. For reference, similar profiles have been computed from minimum morning and maximum afternoon w (red dashed lines). Times of local sunrise and sunset are followed (to within errors) down to about −650 m; for z<−650 m the times between minima and maxima for both d(dI) and w decrease roughly linearly with increasing depth; at large depths (z<−1400 m) errors become large, especially for w for which values become unrealistic.

At the equator, seasonal solar day length did not vary, as was also confirmed by plankton DVM around −587 m (Fig. 4a). Below −650 m, the plankton determined day length decreased steadily with increasing depth, down to about 7.5 hours or 63% of the solar day length at −1600 m. It was thus seen that deepest plankton were the first to rise. The composite plots of Figures 4b,c also showed that plankton migrated over ranges of about 300 m.

**Figure 5:**
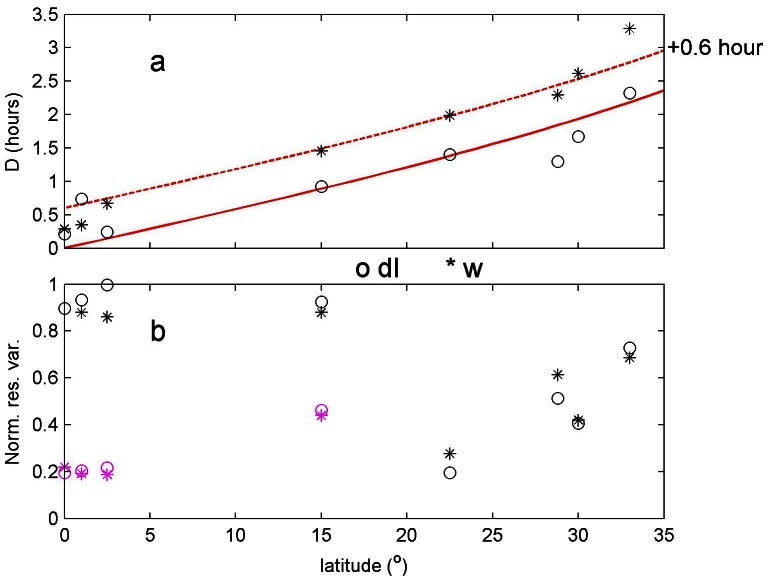
Latitudinal dependence of DVM-amplitude of seasonal variation in day length determined from (derivative of) daily relative dI-values (o, derivative of) and from daily w-values (*) around 1000 m (except 1400 m for mooring LOC113 at 33°N) using harmonic analysis on a one-year periodicity (single frequency of 1/365 cpd) for all moorings in Table 1. (a) Day length amplitudes. The red graph indicates the suns variation in day length according to equation (2). The dashed red line indicates the same graph shifted by 0.6 hours (see text). Note that at 1000 m the average acoustics data residual is 77% (9.3 hours) of the sun's day length. (b) Normalized residual variance, following harmonic analysis. High values are expected near the equator, where a yearlong harmonic is expected to be small. For those data, vertical averages over 200 m were computed and an expected reduced “error” is indicated by purple symbols. Similarly, averages over 4 bins are computed for data from 15°N, which showed unexpected high normalized residual variance.

**Figure 6:**
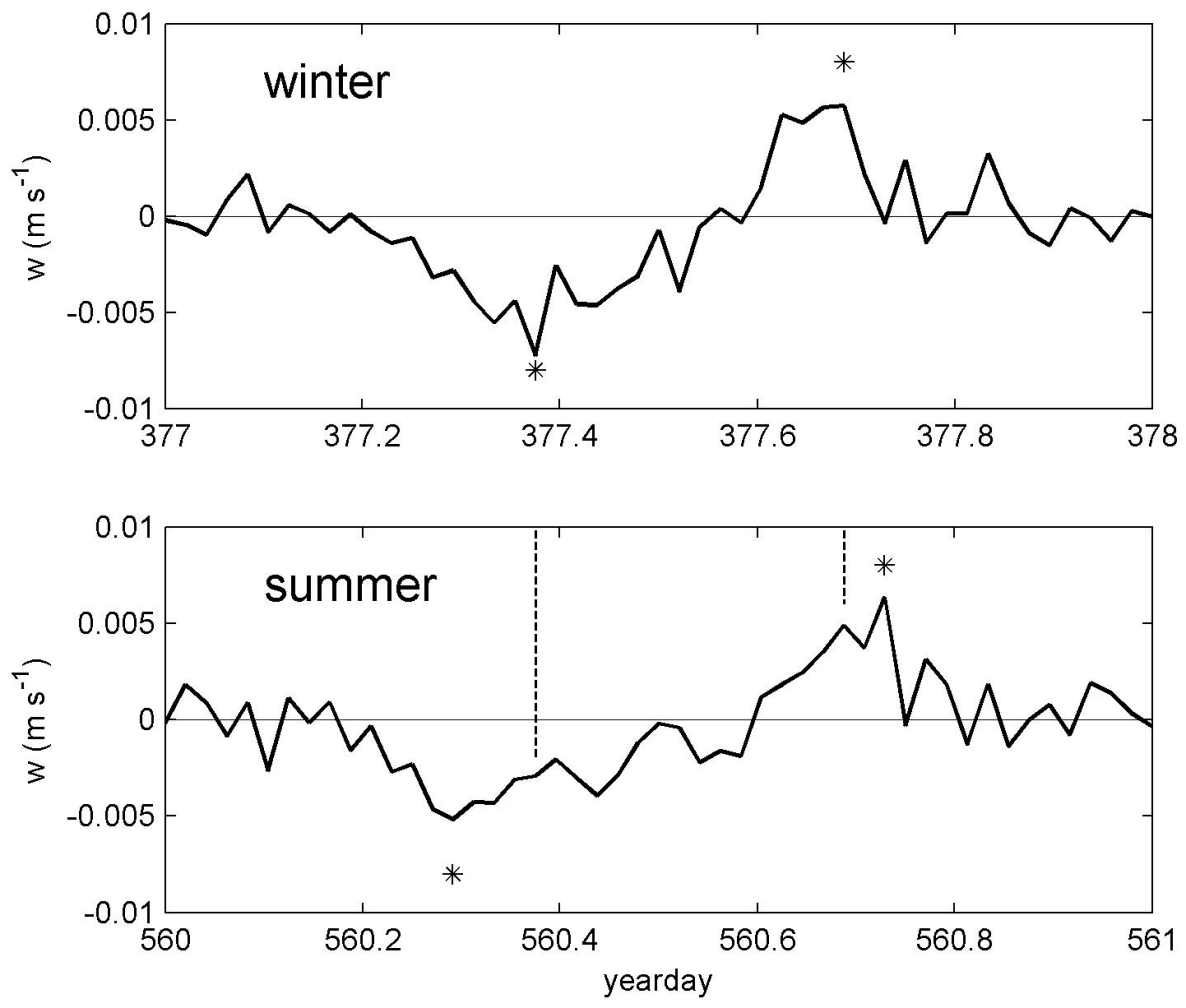
Two typical snapshots of raw one-day period plankton movements (w-data) around −1000 m at mooring LOC164 (22.5°N, 27°W) for winter and summer. In winter, the more diffuse downward migration peaks later than expected as compared with the summer-data. The discrepancy between the absolute time difference between plankton movement (w-peaks) in summer and winter (dashed lines in lower panel) is larger in the morning than in the evening. This explains the larger amplitude of DVM-variations with day length observed in Figure 5 for peak-w (*) by about 40 minutes on average.

When we include all 8 available 1.5 years long time series, the day length variation adopts a latitudinal dependence, which follows model (2) relatively well (Fig. 5a). The main discrepancy between the present observations and the model (2) was a shift by about 0.6 hours for w-data, with the larger amplitude for the latter. This discrepancy could not be explained by equation (2) and it was entirely due to different peak-downward-speeds in winter and in summer (see Fig. 6). In winter, the relatively long period of descent peaks late (day 377.38 in Fig. 6a≈9 o'clock in the day), whereas in summer it peaks early (560.29 in Fig. 6b≈7 o'clock in the day) in the group of downward migrating plankton. No such seasonal variation was found in the group of the more clearly defined ascending plankton (Fig. 6). As sunrise and sunset at 22.5°N both vary over the season by about an hour, the observed zooplankton w-peak in winter was later, by an hour, in the example of Figure 6. Averaged over a year, this extra shift in time amounted to 0.6 hours (Fig. 5). Such extra seasonal differences were not found for both the down- and upward motion periods in dI-data. Note that twice the above extra shift (2×0.6 = 1.2 hours) in w-data did not explain the deviation from solar day length by the acoustic day length means, which were on average decreased to 77% of 12.1 hours (a difference of 2.8 hours) at −1000 m. The fit to the day length model (2) with the present acoustic data was remarkable, as the residual variance was quite high (Fig. 5b). Hence the explained variance by the harmonic daily variation was quite low. This was expected for the equator, where the seasonal amplitude was negligible. However, also at other latitudes away from the equator daily variations in plankton constitution and effects by, e.g., internal wave motions, induce considerable “noise” variations.

## Discussion

The present observations show that DVM of zooplankton in the deep sea follows precise solar variations in day length, across different latitudes and thus matches the sun's latitudinal influx variation. Contrary to the conventional paradigm that DVM would cease in areas where visible light does not penetrate, this study, as well as others [15], [16], thus show that plankton continue to migrate where visible light does not penetrate. Interestingly, deep sea plankton always formed dense aggregations during the day but became diffuse closer to the surface during the night. The dense aggregations formed by zooplankton at depth during the day might suggest a form of anti-predation strategy [23], [24], similar to flocking in birds (e.g., [25]).

Up until recently, it was thought that zooplankton migrations were triggered by the absolute and relative changes in downwelling irradiance [26], [27]. But the maximum depth of sunlight penetration that can be sensed by plankton was estimated to be 700 m at sub-tropical latitudes [17]. This was close to the depth of 650 m at which the plankton (from acoustic dI and w) showed a change in mean day length variation, independent of latitude. Above 650 m, here verified acoustically up to 500 m, mean day length for the plankton was equal to the mean solar day length. Below 650 m, verified acoustically down to 1600 m, mean day length variation for the plankton decreased with depth. These acoustical observations suggest that plankton deeper than 650 m do not sense sunlight.

At all latitudes throughout the vertical range studied, the observed vertical migratory speed of the plankton was ∼0.03 m s^−1^, which was maintained over 3 hours (10^4^ s). Hence, their vertical migratory distance was ∼300 m. These values confirmed previous acoustic observations of DVM (e.g., [5], [9], [13]). As a result, plankton living at a daytime depth of 1000 m or deeper will certainly never directly sense daylight and background solar irradiance. Such individuals thus need a trigger other than sunlight to control their vertical migration.

The question remains as to what the precise trigger mechanism of the seasonal (and lunar [16]) modulated DVM is, especially because in this study and others it has been shown that the deepest plankton groups move first, apparently without solar or lunar light ‘Zeitgeber’ (e.g., [28]).

On the one hand, acoustic noise by moving plankton could be a trigger for the descent, whereby plankton at levels higher-up in the water column, those triggered by the sun, are a signal for deeper plankton to migrate downwards. But such a trigger cannot work for the ascent, as the deepest plankton, that are deprived of sunlight, are the first to migrate upwards. On the other hand, deep-sea DVM could be induced by the temporal variation in free-falling food objects like faecal pellets, but it is unlikely, as their drop speeds are too slow (∼0.005 m s^−1^ (400 m day^−1^); [29]–[31]). Such sinking speeds would cause large phase delays in DVM, i.e. by several days.

Alternatively, DVM could be controlled by precise internal clocks, as has been speculated for lunar modulation in DVM [16]. However, this would require a clock imprint or clock learning in earlier life stages when plankton live closer to the surface, like life stages of a number of zooplankton species (e.g., [32]). As follows from the observations, either the daily variation has to be memorized in the case of signaling by plankton higher up. Or when no such signals are received, a complete seasonal cycle needs to be learned, which might seem unlikely. On the contrary, it is known that biochemical oscillators exist that can maintain stable rhythms for months or even years in the absence of a daily trigger [28], [33]. They do so in many organisms including mammals, migratory birds [34] and unicellular [marine] organisms like cyanobacteria and dinoflagellates [28], [35]. If these results are transferrable to zooplankton, it would be challenging to perform experimental work on zooplankton, starting with studying species in flume tanks under varying light conditions. Subsequently, to see if DVM is genetically programmed, one would need to study different life stages.
